# Cost-outcome description of clinical pharmacist interventions in a university teaching hospital

**DOI:** 10.1186/1472-6963-14-177

**Published:** 2014-04-17

**Authors:** James Gallagher, Stephen Byrne, Noel Woods, Deirdre Lynch, Suzanne McCarthy

**Affiliations:** 1 Clinical Pharmacy Research Group, School of Pharmacy, University College Cork, Cork, Ireland; 2 Centre for Policy Studies, University College Cork, Cork, Ireland; 3 Pharmacy Department, Cork University Hospital, Cork, Ireland; 4 HRB Clinical Research Facility at UCC, Mercy University Hospital, Cork, Ireland

**Keywords:** Hospital pharmacy, Adverse drug events, Health care economics, Ireland, Clinical pharmacy services, Cost avoidance

## Abstract

**Background:**

Pharmacist interventions are one of the pivotal parts of a clinical pharmacy service within a hospital. This study estimates the cost avoidance generated by pharmacist interventions due to the prevention of adverse drug events (ADE). The types of interventions identified are also analysed.

**Methods:**

Interventions recorded by a team of hospital pharmacists over a one year time period were included in the study. Interventions were assigned a rating score, determined by the probability that an ADE would have occurred in the absence of an intervention. These scores were then used to calculate cost avoidance. Net cost benefit and cost benefit ratio were the primary outcomes. Categories of interventions were also analysed.

**Results:**

A total cost avoidance of €708,221 was generated. Input costs were calculated at €81,942. This resulted in a net cost benefit of €626,279 and a cost benefit ratio of 8.64: 1. The most common type of intervention was the identification of medication omissions, followed by dosage adjustments and requests to review therapies.

**Conclusion:**

This study provides further evidence that pharmacist interventions provide substantial cost avoidance to the healthcare payer. There is a serious issue of patient’s regular medication being omitted on transfer to an inpatient setting in Irish hospitals.

## Background

The traditional role of a pharmacist predominantly involved the dispensing of medications in both hospital and community settings; consequently the pharmacist was quite detached from other healthcare professionals. The profession has since evolved to become recognised as an essential part of the healthcare team [1]. While still ensuring that medicines are sourced and dispensed to the highest possible standards, pharmacists have diversified into alternative areas of care in hospital practice [2]. Interventions are integral components of the new enhanced role which pharmacists offer in a clinical setting [3-8].

A pharmacist intervention is defined as any action taken by a pharmacist that aims to change patient management or therapy [9]. A pharmacist’s expertise in pharmacology, pharmacotherapy and pharmaceutics ensures they have the requisite capabilities to offer suggestions to other healthcare staff on possible alterations to a patient’s therapy [10,11]. This helps to ensure optimal patient outcomes, which has the potential to have an add-on economic benefit to the healthcare institution.

A myriad of studies have described the high rate of potential inappropriate prescribing and potential adverse drug events (ADE) in multiple healthcare systems [12-16]. An ADE is defined by the International Conference for Harmonisation as “any untoward medical occurrence in a patient administered a medicinal product which does not necessarily have to have a causal relationship with treatment”. These issues cause repercussions in the form of increased resource utilisation [17]. Evidence of the clinical benefit and reduction in ADEs associated with enhanced roles for pharmacists in a hospital setting, are documented in the literature [2,5,18,19].

Healthcare systems worldwide are coming under increasing pressure due to a combination of aging populations and the proliferation of new expensive technologies [20]. All healthcare services need to show that they provide value for money for the investment made in their provision [21]. Despite early scepticism, health economic evaluations are required for the establishment and continued provision of services and technologies [20].

The provision of a clinical pharmacy service in a hospital setting is an investment which utilises costs which could be used elsewhere in the health system. Economic evaluations of clinical pharmacy services will help policy makers make informed decisions on whether they are a worthwhile investment. Studies in other jurisdictions have indicated that they are cost-effective, however these findings are not generalisable [22,23].

This paper will analyse the interventions made by a team of pharmacists in a university teaching hospital and evaluate the cost avoidance achieved through the prevention of an ADE. For the purposes of this study, cost avoidance refers to an intervention that reduces or eliminates additional expenditure that otherwise may have been incurred in the absence of the intervention [24]. It is a different measure to cost saving interventions, which refer to reductions in current spending due to changes in the expenditure on a patients treatment [25]. Cost avoidance interventions contain and control costs and over a longer period of time they can result in cost savings.

The study was based in an Irish university teaching hospital/tertiary referral centre setting over a period of one year. Similar studies have been performed, examining shorter time periods or focusing on specific interventions [3-7,25]. However, information which helps to evaluate the economic impact of pharmacist interventions over a longer period of time and covering an entire hospital domain is lacking. The authors have been unable to find a study where cost avoidance generated by a full department of clinical pharmacists in a full calendar year has been calculated.

## Methods

### Setting

This was a retrospective study based on a 1 year time period from 01/01/2012 to 31/12/2012 inclusive. Cork University Hospital (CUH) and the Cork University Maternity Hospital is a combined 850-bed hospital site. The hospital serves a population of over 620,000. In addition, it is a tertiary referral centre for over 1 million people, in the southern region of the Republic of Ireland. Approximately 32,000 inpatient admissions were recorded for 2012. All patients who were in receipt of a pharmacist intervention on their drug therapy were included in this study. There were no additional exclusion criteria. The Pharmacy Department consists of 15.8 whole time equivalent (WTE) pharmacists. As two WTEs are employed in managerial and administrative capacities, 13.8 WTE are available to document interventions as part of the daily pharmaceutical service. Interventions performed in all areas of the hospital, including the maternity unit, were included in this study.

### Intervention analysis

Clinical pharmacist interventions are provided by many basic and senior grade pharmacists at CUH. Clinical pharmacist interventions are carried out at patient admission, during pharmacist led patient chart review or at the request of another healthcare professional. As previously discussed, the primary goal of a clinical pharmacist intervention is to ameliorate patient therapy.

Interventions made by pharmacists were recorded on a duplicate paper form. One form is kept by the pharmacist and one is passed on to the attending physician who has the final decision on whether to accept or reject the intervention.

In addition to producing a paper record of the intervention, the pharmacist retrospectively enters the proposed intervention into the ‘eClinical Pharmacy Suite’. The ‘eClinical Pharmacy Suite’ is a browser-based application, aimed at supporting clinical pharmacists to record, grade and report medication related interventions or errors. Interventions were assigned by the clinical pharmacist to the most appropriate category in the application. Due to time constraints, not all recorded intervention had been entered on the computerised database. The primary researcher inputted any outstanding interventions and verified data which had been previously entered by hospital pharmacists. Intervention categories were developed based on the recommendations of an advisory group of clinical pharmacists from Ireland.

### Cost analysis

The cost of providing this service was calculated based on the average time it took to carry out an intervention and the hourly cost of employing a hospital pharmacist. The average time of an intervention was based on a previously published study, which showed that the majority of pharmacist interventions in a university hospital setting took between 15 – 30 minutes to complete [7]. The hourly rate of employing a pharmacist at the mid-point of the salary scale was calculated based on an annual salary of €49,425 (Point 6 of 2010 salary scale) [26]. This underwent upward revision to account for employer related costs and hospital overheads based on guidance for conducting an economic analysis within the Irish healthcare system [27-29]. Base case scenario was calculated using the hourly cost of employing a basic level pharmacist at the mid-point of the salary scale and an average intervention time of 22.5 minutes.

Cost avoidance was calculated based on the probability that an ADE would have occurred in the absence of the proposed pharmacist intervention [18]. Interventions were analysed by the primary author and a score was assigned based on the probability of a patient experiencing harm directly or indirectly from their prescribed/administered medicines, and also the potential of omission of regular medication, sub-therapeutic dosing or an ill-advised choice of therapy. Determination of the probability that a patient would experience harm in the absence of an action by a pharmacist was based on the methodology described by Nesbit et al. [25] (Table 1). The cost avoidance for each individual intervention was determined and total cost avoidance was accordingly calculated through summation of the individual interventions. A random sample of the interventions (n = 100) were reviewed by two academic pharmacists with hospital pharmacy experience and inter-rater reliability was calculated for the sample.

**Table 1:** **Nesbit method for calculating indirect cost benefit**[25]

**Equation 1: Cost avoidance for individual intervention**	***Probability of an ADE occurring X cost of an ADE *** [30] ***OR (0 or 0.01 or 0.1 or 0.4 or 0.6) X €1057***	
**Probability of an ADE occurring**	**Probability score**	**Example**
No harm expected	0.00	Pharmacist suggests changing patient from esomeprazole to omeprazole exclusively for economic reasons.
Very low	0.01	Patient regularly takes a bisphosphonate, but medication omitted from hospital kardex
Low	0.10	Patient takes an antibiotic twice daily, when recommended dose would be three times daily.
Medium	0.40	Metformin dose not reduced despite patient demonstrating renal impairment.
High	0.60	Patient prescribed amiodarone while currently taking digoxin without any reduction in digoxin dose.

*Source* Nesbit, T.W. et al.: Implementation and pharmacoeconomic analysis of a clinical staff pharmacist practice model. American journal of health-system pharmacy 58(9), 784–790 (2001).

The authors were unable to find an estimated cost of an ADE, calculated based on data from the Irish healthcare system. The additional cost of treating an inpatient that experienced an ADE was taken from a recent study which utilised a micro-costing approach based on data from German hospitals [30]. This study also included a range of previously published ADE estimates (€934 – 5783). The majority of previously published studies which calculate cost avoidance used a cost of ADE determined by Bates et al. [5,7,24,25]. Rottenkolber ADE valuation was deemed to be more appropriate for this study as it was published in 2012 while Bates study was published in 1997 using US data [31]. This removed the need to account for currency differentials and inflation. Purchasing power parities between Germany and Ireland were used to further minimise differentials between the two countries. Cost of an ADE used in base case scenario was €1057.

A micro-costing approach, assigns a valuation to each individual unit of resource consumed and is considered the most robust costing method [32]. Diagnosis related group (DRG) costs for toxic side effects of drugs based on Irish hospital data were available but were not chosen as the cost of an ADE. The DRG estimate exclusively measured toxic side effects of drugs [33], furthermore DRG costs are generally less accurate in comparison to micro-costing estimates [32]. DRG costs for toxic side effects for drugs (€887) were included in sensitivity analysis calculations.

Following estimation of the cost of carrying out the pharmacist interventions and the resulting cost avoidance, net cost benefit and cost benefit ratio for providing the service were calculated. Analysis was calculated from the perspective of the healthcare institution. Discounting was excluded as events were all considered to have taken place in a 1 year time period.

### Sensitivity analysis

One way deterministic sensitivity analysis was performed. Published ranges and confidence intervals where available determined the extent of the parameters. Sensitivity analysis was also performed using alternative published costs of an ADE [31], Irish DRG data and with various intervention acceptance rates.

### Data analysis

Reports generated from the eClinical Pharmacy Suite database were in Microsoft Excel^TM^ format. Summary statistics were calculated through Microsoft Excel 2010 (Microsoft Corp., Redmond, Washington). All other advanced analysis was conducted through IBM SPSS Statistics Version 18.

### Ethical approval

Approval was obtained from the Clinical Research Ethics Committee of the Cork Teaching Hospitals, University College Cork, Ireland.

## Results

A total of 4,257 interventions were documented on 2,147 individual patients (Table 2). The majority of the interventions were judged to have prevented potential ADEs (n = 3,417). The remaining interventions had no discernible impact on therapy or patient outcomes, based on the judgement of the primary author. Additional interventions required entry under multiple categories on the database, but were only evaluated once for potential prevention of an ADE.

**Table 2:** Intervention analysis

	**Number of interventions**
Total number of patients who received intervention	2147 patients
Total number of interventions	4257
Mean number of interventions per patient (St. dev.)	1.98 (1.60)
Median number of interventions per patient (95% CI)	1 (0.06)
Range of interventions per patient	1 - 18
Interventions accepted by physicians (%)	1275 (29.92)
Interventions rejected by physicians (%)	61 (1.43)
Interventions with unknown acceptance outcome (%)	2921 (68.81)

Recorded acceptance rate by physicians was 29.92% (n = 1275). Only 1.43% (n = 61) interventions were recorded as being rejected by a physician. However, the rate of interventions with an unknown acceptance outcome was high, 68.81% (n = 2921).

Substantial cost avoidance of €710,000 was generated over a 1 year period from the perspective of the health care provider. Mean cost avoidance of €166 per intervention was generated. The cost of providing these interventions was €82,000. Substantial net cost benefits of €626,279 and a cost benefit ratio of 8.64 were generated based on this evaluation of pharmacist interventions (Table 3).

**Table 3:** Cost analysis of pharmacist interventions

		**Base case**
		**(Range)**
1.	Cost avoidance	**€708221**
		(625808–3874783)
**2.**	**Cost of Service**	
	- Pharmacist Wages	**€81942**
		(22402 –110389)
^**a**^**3 = (1–2)**	Net Cost Benefit	**€626279**
		(567173–3764394)
^**b**^**4 = (1/2)**	Cost Benefit Ratio $\frac{\mathrm{Net}\;\mathrm{Cost}\;\mathrm{Benefit}}{\mathrm{Cost}\;\mathrm{of}\;\mathrm{Service}}$	**8.64**

^a^(Cost avoidance – Cost of Pharmacy Services).

^b^(Net Cost Benefit ÷ Cost of Service).

The number of interventions that potentially avoided ADEs were as follows: 119 (2.8% of all interventions) of the interventions were associated with a probability score of 0.6 (high likelihood of preventing an adverse event), 1101 interventions (25.86%) were associated with a probability score of 0.4 (medium), 1514 interventions (35.56%) were associated with a probability score of 0.1 (low), 683 interventions (16.04%) were associated with a probability score of 0.01 (very low) and 840 interventions (19.73%) were associated with a probability score of 0 (no harm expected).

The most prevalent type of intervention was the identification of omissions of patient’s regular pre-admission medication, followed by requests to change the dose of medications and requests for the physician to consider whether it was appropriate to continue with a medication (Table 4). The most common categories of medications to require interventions were proton pump inhibitors (n = 259), statins (n = 208), beta-blockers (n = 165), corticosteroids (n = 161) and penicillins (n = 157).

**Table 4:** Intervention categorisation

**Category of intervention**	**N (%)**
Drug	2759 (64.81%)
*- Drug, Omissions*	1820 (65.93% of Drug Category)
*- Drug, Review Therapy*	421 (15.24% of Drug Category)
*- Drug, Interaction*	124 (4.56% of Drug Category)
*- Drug, Other*	394 (14.27% of Drug Category)
Doses	920 (21.61%)
Frequencies	354 (8.32%)
Routes	125 (2.94%)
Duration	47 (1.10%)
Other	27 (0.63%)
Date/Time	20 (0.47%)
Rates	5 (0.12%)

Inter rater reliability indicated an acceptable level of agreement, based on a random sample of 100 interventions. Average agreement between 3 raters was 0.744. Individual pairwise agreement was over the significant level of 0.7 for all 3 comparisons: Primary Rater (PR) – Academic Pharmacist (AP) 1 = 0.763, PR – AP 2 = 0.761, AP 1 – AP 2 = 0.709.

In all scenarios examined, the cost-benefit ratio remained positive (Table 5). All known variables underwent a one-way sensitivity analysis based on known ranges or using variations used in previously published sensitivity analysis on the topic. Nesbit et al. conducted a sensitivity analysis where the ADE probability underwent an each way variation of 50%, an identical variation was undertaken in this paper [25]. The greatest variance in cost-benefit ratio was displayed in the cost assigned to an ADE.

**Table 5:** Sensitivity analysis for cost-benefit ratios

**Variable**	**Lower limit *(Cost Benefit Ratio)***	**Upper limit *(Cost Benefit Ratio)***
Time	**30 minutes per intervention** 6.48	**15 minutes per intervention** 12.96
ADE Probability	**-50% Probability score** 4.32	**+50% Probability score** 12.96
Salary	**Highest point on senior pharmacist scale** 6.42	**Lowest point on basic pharmacist scale** 12.07
ADE Cost^A^	**Lowest point on range** 7.63	**Highest point on range** 47.28
Intervention acceptance	**50% Acceptance** 4.32	**Known Acceptance (29.92%)** 2.59

^A^ADE range taken from a review of selected international studies regarding the economic consequences of ADEs which reported additional mean costs in the range of €934 to €5783 per case [34].

Two additional ADE cost estimates were investigated during the course of sensitivity analysis (Table 6). The estimated cost of an ADE calculated by Bates and adjusted due to change in setting and year resulted in a considerable increase in the cost benefit ratio.

**Table 6:** Sensitivity analysis using alternative ADE estimates

**Alternative ADE estimate**	**Cost benefit ratio**
DRG Toxic Side Effects [33]	7.25
Bates ADE Costing [31]	49.49

## Discussion

Substantial cost avoidance was demonstrated in this study. Cost benefit ratio and net cost benefit remained positive under all conditions examined. To the authors’ knowledge, this study is the first that has attempted to estimate the cost avoidance achieved in a total hospital environment over such an extended period of time. Direct comparison of savings generated in this study with previously published studies is difficult. The generalizability of pharmacoeconomic analysis is uncertain [35]. Calculation of cost avoidance will have inter study variations in the cost assigned to an ADE, methodologies, healthcare settings, duration of study and number of pharmacists employed.

The original study which implemented the cost avoidance method used in this paper generated a net benefit of $392,660 [25]. This was over a similar 12 month period but only included 3 WTE pharmacists operating on three specific hospital wards. Study location was in a US hospital. These pharmacists had undergone specialist training and were to a large extent exclusively performing interventions during their participation in the study. Cost benefit ratio of 3.1:1 was considerably smaller in the Nesbit study. Cost-benefit ratio was influenced significantly by the method used to calculate the cost of the service. The complete pharmacist salary was used to calculate the cost of providing the intervention rather than apportioning part of the salary based on the time pharmacist spent enacting the interventions.

A study by Olson et al. conducted in a 360 bed US hospital has the greatest methodological agreement with this paper [7]. This was conducted over a 3 month period and estimated the cost benefit ratio to be 1.2. Only 5 pharmacists provided interventions in this study. Although, substantial savings ($84,631) was generated from a small number of interventions, the cost-benefit ratio was lower than expected. As with the Nesbit study, cost-benefit ratio was reduced as full pharmacist salary was used to calculate the cost of providing the service. The results in this study provides evidence that the positive cost benefit ratio of clinical pharmacists’ interventions is maintained over a longer period of time, with additional pharmacists and in a wider hospital setting.

Cost avoidances are impacted significantly if they are focused in specific departments. Cost avoidance per intervention generated in our study was decidedly lower in comparison to a study conducted in an intensive care unit. The addition of a single critical care pharmacist to a 16 bed intensive care unit produced an average cost avoidance of $1596.27 - $1615.67 per intervention [5]. Input costs were omitted in this study so further comparison was not feasible.

While the cost-benefit ratio in this study was positive, it needs to be reiterated that this ratio was based on estimates of time and avoidance of cost rather than hard economic data. Therefore, the ratio could potentially be an overestimate. Furthermore, an evaluation of a clinical pharmacy service is strengthened when it also includes an assessment of the clinical and humanistic outcomes involved [36].

This high degree of omissions of patient’s regular pre-admission medications (43% of all interventions) highlights the need for a dedicated system of medicines reconciliation at a tertiary healthcare level in Ireland. A pilot study of pharmacist-led medication reconciliation in two university teaching hospitals, elsewhere in Ireland, also identified omission of a pre-admission medication as the most common discrepancy [37]. These findings are replicated in other healthcare settings [38]. Medication omissions can have potentially serious consequences for patients depending on the nature of the drug omitted [39].

An argument could be presented not to include medication omissions as an ADE, based on a strict interpretation of the definition, as the patient did not receive the drug [40]. However, the decision was taken to include them in this study. Similar studies in the past have included the identification of medication omissions as a potential ADE [7,41]. The probability of a patient incurring harm is increased if they do not receive their regular medication, resulting in a related increase in hospital resource utilisation [42].

Medium and low scores were the most frequent probabilities assigned to the interventions. This reflects findings obtained in the majority of previous studies which implemented the same method of calculating cost avoidance [5,25,41]. The frequency of these scores were influenced by the number of medication omissions (39% of omissions were assigned a medium probability score and 37% assigned a low probability score.) Interventions designated with a high probability of preventing an ADE were largely composed of omissions of essential medications (e.g. anti-epileptic drugs) or known serious drug interactions.

The most common medication classifications requiring intervention were unsurprising. Proton pump inhibitors (PPI) were the medication classification requiring the most frequent intervention. Interventions on PPIs were largely suggestions to change to lower cost equivalents, highlighting therapeutic duplication, excess of therapy or suggestion for switching from intravenous to oral administration. The majority of these interventions resulted in cost savings to the healthcare provider. Inappropriate prescribing of PPIs is a significant issue in the Irish healthcare system and a significant drain on resources [43,44]. This demonstrates the pivotal role can play as cost containers in the healthcare system.

Another method of increasing the number of interventions would be the provision of further training for clinical pharmacists which would enable them to become specialists in various areas of patient care. This practice is common in other countries and has been shown to provide substantial monetary savings from interventions enacted by specialist pharmacists [5]. Specialised anti-microbial pharmacists have been shown to be of value and are now established in many hospitals in Ireland [45,46].

A significant level of agreement was found between three of the listed authors. Assignment of probabilities is subjective; therefore a high level of agreement is unlikely. On review of samples, almost all were within 1 score of each other, further indicating that probabilities were assigned by the primary author in a manner consistent with fellow professionals. Although, inter rater reliability was not examined for all interventions, the clinical background of the primary rater and the significant agreement level demonstrated in the sample indicate the ADE probabilities were assigned in an appropriate and consistent manner.

There was a high rate of interventions where the outcome was unknown at time of analysis. However, review of interventions where status was determined indicated that only a small minority of pharmacist interventions were rejected. This indicates that other healthcare professionals are receptive to pharmacist interventions on patient medication. The high level of unknown outcomes was most likely due to time constraints on the pharmacist. Following acceptance or rejection, system requires manual updating. It is understandable that an administrative task such as this may be neglected.

The importance of an accurate estimation of ADE cost was emphasized from the dramatic increase in cost benefit ratio if Bates et al. estimate of ADE was used. As previously stated, there is an absence of data on ADE costs in Ireland. The validity of estimated cost avoidance would be improved through application of local data on excess costs associated with ADE. Until this issue has been addressed, imperfect data is the only viable option. Sensitivity analysis undertaken in this study was deterministic in nature; performing probability sensitivity analysis would have been a more accurate robust method to determine whether pharmacist interventions would have maintained a positive cost benefit ratio.

The primary limitation was the limited availability of additional patient information (medical record, medical history, outcome of intervention etc.) when assigning adverse drug event probabilities. As this study contained a large number of patient interventions, it was not feasible to retrospectively retrieve this information from medical notes.

Another major limitation was the exclusion of some potential input costs. In order to conduct an intervention, problems must be located which takes up pharmacist time and therefore has an associated cost. Screening of a patient’s medication may not be exclusively for the purpose of discovering interventions but it is one possible outcome from it. Additionally, other healthcare professionals are required to spend time reviewing suggested pharmacist interventions. This was also excluded from analysis.

Utilisation of a scoring system which also accounts for the severity of the potential ADE would significantly enhance this study. Such scoring systems exist but they do not assign a cost with the scoring outcome generated. There is no ideal system for assigning probabilities to adverse events but the widespread adaptation of one system would add to the ability to compare studies across jurisdictions. The classification of interventions is subjective. Generation of local guidelines on the classification of interventions would help reduce this variation.

While the interventions included in the study represented the majority of work conducted by clinical pharmacists, it is possible that some interventions were not inputted on to the eClinical System. Therefore, the current data may under-represent the cost avoidance produced.

The interventions were assigned scores by an individual rater. While sample of interventions examined for inter-rater reliability indicated that the primary author was assigning probability scores in a manner consistent with other pharmacists, review of complete dataset by additional pharmacists and other medical professionals would have enhanced the study.

## Conclusion

Previous reviews have indicated that pharmacist interventions generate significant cost avoidance when measured under certain criteria and conditions [18]. This study has confirmed previous opinions and supplemented the body of evidence that the provision of clinical pharmacy services in an entire hospital provides value for money to the healthcare payer. An excessive amount of omissions of regular medications has been highlighted by this study. The estimation of cost avoidance would be improved by the development of a method which incorporated the potential severity of an ADE into the evaluation and an evaluation of excess costs associated with ADEs in an Irish healthcare setting.

## Competing interests

The authors declare that they have no competing interests.

## Authors’ contributions

JG performed analysis on data and was the primary author of the manuscript. SB devised the concept for the manuscript, performed analysis on sample data and revised drafts of the manuscript. NW provided advice on appropriate methods for calculating costs and revised drafts of the manuscript. DL revised drafts of the manuscript. SMcC participated in data collection, performed analysis on sample data and revised drafts of the manuscript. All authors read and approved the final manuscript.

## Pre-publication history

The pre-publication history for this paper can be accessed here:

http://www.biomedcentral.com/1472-6963/14/177/prepub
